# Lucky Rhythms in Orbitofrontal Cortex Bias Gambling Decisions in Humans

**DOI:** 10.1038/srep36206

**Published:** 2016-11-10

**Authors:** Pierre Sacré, Matthew S. D. Kerr, Kevin Kahn, Jorge Gonzalez-Martinez, Juan Bulacio, Hyun-Joo Park, Matthew A. Johnson, Susan Thompson, Jaes Jones, Vikram S. Chib, John T. Gale, Sridevi V. Sarma

**Affiliations:** 1 Department of Biomedical Engineering, Johns Hopkins University, Baltimore, MD 21211, USA; 2 Center for Epilepsy, Neurological Institute, Cleveland Clinic, Cleveland, OH 44106, USA; 3 Department of Neuroscience, Lerner Research Institute, Cleveland Clinic, Cleveland, OH 44195, USA; 4 Center for Neurological Restoration, Neurological Institute, Cleveland Clinic, Cleveland, OH 44195, USA.

## Abstract

It is well established that emotions influence our decisions, yet the neural basis of this biasing effect is not well understood. Here we directly recorded local field potentials from the OrbitoFrontal Cortex (OFC) in five human subjects performing a financial decision-making task. We observed a striking increase in gamma-band (36–50 Hz) oscillatory activity that reflected subjects’ decisions to make riskier choices. Additionally, these gamma rhythms were linked back to mismatched expectations or “luck” occurring in past trials. Specifically, when a subject expected to win but lost, the trial was defined as “unlucky” and when the subject expected to lose but won, the trial was defined as “lucky”. Finally, a fading memory model of luck correlated to an objective measure of emotion, heart rate variability. Our findings suggest OFC may play a pivotal role in processing a subject’s internal (emotional) state during financial decision-making, a particularly interesting result in light of the more recent “cognitive map” theory of OFC function.

The decisions we make on a daily basis are heavily influenced by our emotions. Phenomena like loss aversion or risk sensitivity can bias decisions away from maximization of expected reward^1^,^2^. Emotional influences can also be seen through more superficial biases, like that of an attractive face^3^. From a behavioral perspective, ways that emotions can bias our decisions have been extensively studied (refs 4, 5, 6 to name a few). However, the specifics of how these biases are represented in the brain are still not well understood. Considerable physiological and anatomical evidence suggests that the OrbitoFrontal Cortex (OFC) is involved in both decision-making and emotional processing.

Activity in the OFC is associated with value encoding of choices^7^,^8^, stimuli^9^, discrepancies between realized and expected results^10^, and compulsive decision-making^11^. In addition, lesions of the OFC have been shown to influence a person’s ability to perform reversal learning^12^,^13^ and to impair the ability to incorporate emotional cues into his or her decisions^14^. Other lesion studies, such as those in macaques^15^, support the idea the OFC helps assign credit for outcomes to previously made decisions^9^,^16^,^17^. Single unit work in rats further indicates a strong link between OFC and stimulus-outcome learning over action-outcome learning^18^.

While a diverse set of explanations have arisen to explain the extensive body of OFC related work, recently studies have emerged supporting the idea OFC functions as a sort of “cognitive map”^19^. That is, the OFC maintains or provides key information for a state-space description of a task and reflects the organism’s location within it, particularly aspects that are not reflected in the environment. Single unit recordings^20^ and other neuropsychological data^21^ indicate the OFC contains complex information regarding internal choice allowing the disambiguation of states that seem similar externally but result in different outcomes. In this paper, we present data suggesting that OFC activity contains information dissociating between the choices of two equivalent options in the face of identical external stimuli, reflecting a bias stemming from an internal state—possibly an internal emotional state.

We accomplished this by analyzing local field potentials recorded directly from the human OFC during a gambling task as a means of examining relationships between OFC activity, inferred emotional state, and decision-making. The OFC is typically studied using human case studies, functional Magnetic Resonance Imaging (fMRI) in humans, lesion experiments in animal models, or electrophysiology in monkeys or rats. Typical concerns associated with interpreting fMRI studies are the poor temporal resolution and the blood oxygenation level-dependent signal only being a proxy for neural activity^22^. On the other hand, conclusions drawn from rat and monkey studies suffer from difficulties in translation to humans^23^. By taking advantage of Stereotactic ElectroEncephaloGraphy (SEEG) recordings in epileptic patients at the Cleveland Clinic^24^, we were able to examine local field potential activity of the OFC in five humans at high temporal resolution (1 or 2 kHz). With this technique, we can both analyze quick neural changes as well as draw straightforward conclusions for human OFC function. While OFC activity has always been studied in response to a stimulus or reward, we are able to show that gamma rhythms in OFC present *before* any stimulus is shown may influence behavior. We are further able to link these gamma rhythms back to the subjects’ heart rate variability (HRV)—a proxy to emotion—and mismatched expectations in past trials, supporting the idea the OFC reflects the current state with respect to the task using internal information.

Patients implanted with SEEG for seizure localization had the opportunity to participate in this decision-making study^25^. All patients in our study signed a Cleveland Clinic Institutional Review Board approved informed consent before participation. Five subjects volunteered who happened to have SEEG electrodes in OFC. The task involved a gambling decision (Fig. 1a). Subjects played a game of high card where they would win virtual money if their card beat the computer’s card. Specifically, in the beginning of each trial, the subject controls a cursor via a planar manipulandum (Interactive Motion Technologies, USA) to a fixation target. Afterwards, subjects are shown their card^2,4,6,8^ or^10^ that is randomly chosen with equal distribution (known by the subjects). The computer’s card is initially hidden. The screen then shows their two choices, a high bet ($20) and a low bet ($5). The subject has 6 seconds to select one with their cursor. Following selection, the computer’s card, which follows the same distribution, is revealed. The final screen depicts the amount won or lost. See *Subjects* and *Gambling Task* in *Methods* for more details.

## Results

**Figure 1:**
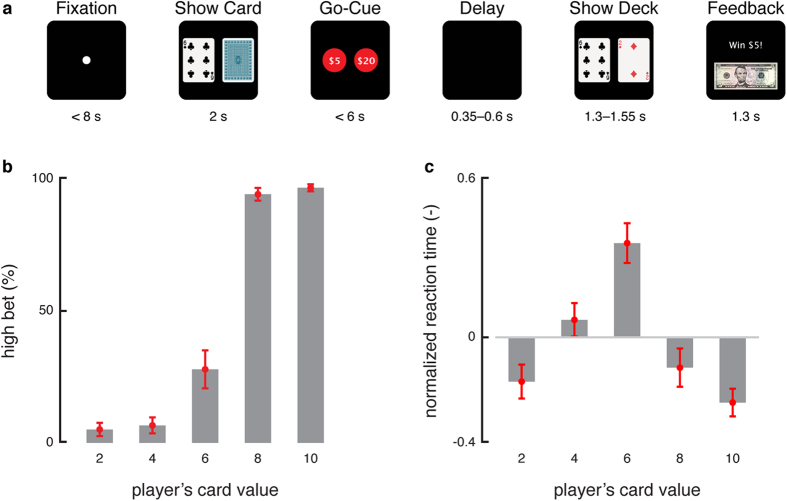
Gambling task and behavioral results. (**a**) Timeline of the behavioral task. After fixation, subjects were shown their card. Once the bets were shown, subjects selected one of the choices and then were shown the computer’s card following a delay. Feedback was provided afterwards by displaying the amount won or lost. (**b**) Average bet decisions across cards. Subjects predominantly bet low for 2 and 4 cards and bet high for 8 and 10 cards. There was no predominant strategy for 6 cards, which had a 29% chance of eliciting a high bet. (**c**) Average normalized reaction times across cards. Subjects reacted slower for cards whose rewards had higher variability. Error bars represent one standard error of the mean.

The optimal strategy for maximizing expected reward in this task is straightforward: bet high when dealt the 8 or 10 card and bet low when dealt the 2 or 4 card. Subject behavior closely followed this strategy (Fig. 1b). In contrast, for the 6 card, both betting options resulted in the same expected reward. The ambiguity of the decision on 6-card trials resulted in subjects switching between both high and low bets and taking longer to decide (Fig. 1c).

For their decisions, subjects on average bet high 29% of the time for these 6-card trials, thus exhibiting risk-averse behavior. Given that a decision must be made despite both decisions having the same expected reward, decisions are likely based on some internal bias. We hypothesize that the choice on each 6-card trial is driven by the subject’s internal state that fluctuates on a trial-by-trial basis and that this internal bias is mediated by the OFC.

### Linking OFC to an internal bias that predicts future behavior

**Figure 2:**
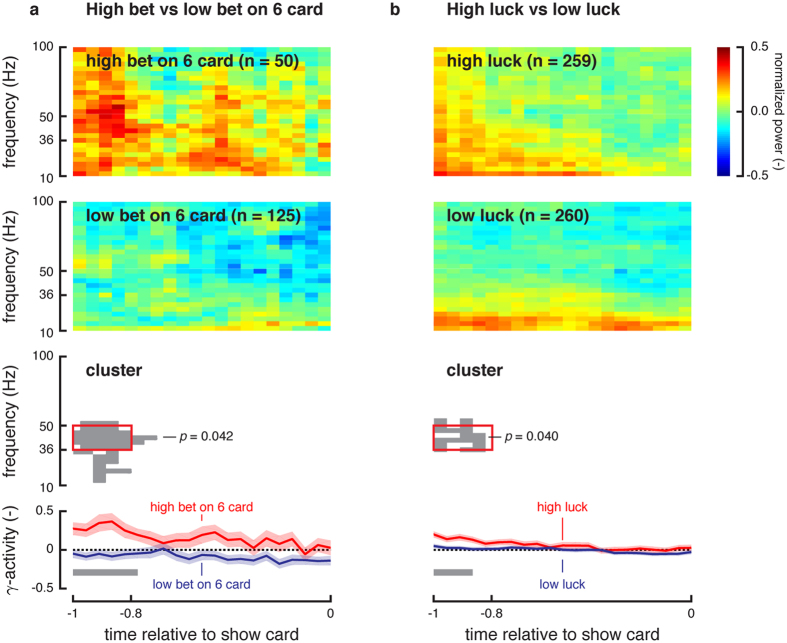
Oscillatory power before the Show Card. (**a**) The average spectrograms show differences between high-bet and low-bet conditions on 6-card trials. One significant cluster (*p* = 0.042) resulted from the cluster-based nonparametric statistical test. The cluster contained frequencies between 36 and 50 Hz at a timing between 1000 and 800 ms before the Show Card. This frequency range matches the traditional lower gamma band. Plots of average oscillatory power (36–50 Hz) over time for 6-card trials resulting in high and low bets show the modulation of the power in the gamma band preceding the Show Card. Time bins with significant differences are marked by the grey bar. Error bars represent one standard error of the mean. The number *n* denotes the number of trials pooled across patients. (**b**) The average spectrograms show differences between high-luck and low-luck conditions on all trials. One significant cluster (*p* = 0.040) resulted from the cluster-based nonparametric statistical test. The cluster is located in the similar time-frequency region as the cluster emerging from the high-bet and low-bet conditions on 6-card trials.

We first tested the link between gambling decisions on these 6-card trials (i.e., trials with the greatest uncertainty of a prospective win) and oscillatory power in OFC local field potentials. Oscillatory power was used due to its common association with synchronized activities of the underlying neuronal population encoding behavior^26^. See *Spectral Analysis* in *Methods* for more details concerning the calculation of oscillatory power^27^. To ensure bias encoding instead of decision encoding, neural oscillations were analyzed *before* subjects even saw their card at the beginning of each trial. To determine whether any OFC activity correlated to betting decisions, OFC oscillatory power was compared between the set of trials where subjects end up betting high on a 6 card and the set where they end up betting low. The average normalized spectrograms for both high and low bet trials show that high bet trials have higher 40–50 Hz oscillatory power about 1000 milliseconds (ms) preceding the show card epoch (Fig. 2a). To determine statistical significance of this effect, we used a cluster-based nonparametric statistical test^28^. Clusters are defined as a set of adjacent time-frequency windows whose activity is different between trials where the subjects end up betting high versus low. See *Cluster-Based Nonparametric Statistical Test* in *Methods* for more details.

The most significant cluster emerged between 36–50 Hz around 1000 and 800 ms before the card is shown (*p* = 0.042). It had significantly higher power during 6-card trials where the subjects ended up betting high (Fig. 2a, third panel). Comparing the average gamma power, as defined by this frequency range, shows that the power is significantly higher 1000 to 800 ms *before the subject even sees the 6 card* on trials where the subject ends up betting high (Fig. 2a, fourth panel). Additionally, we tested if the modulation of OFC oscillatory power in the same time period was merely a result of other effects, including effects from outcome or reward processing in the previous trial. No significant clusters were found (see data in Supplementary Information). Future references to “OFC activity” in the remainder of this report relate to the average power in the cluster defined by 1000–800 ms before the show card and 36–50 Hz (red rectangle in Fig. 2a, third panel).

The influence of OFC activity was then assessed for each card type individually by constructing a model of the probability of betting high on a given card. This test was performed to see if this OFC activity improves prediction of betting behavior on any card other than the 6-card trials, and the model will be compared to models constructed in remaining analyses below. Using a logistic function to ensure probability values between 0 and 1, the subject’s betting behavior was modeled as:


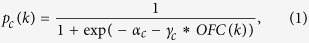


where *p*_c_(*k*) is the probability of betting high on card *c* = {2, 4, 8, 10} for trial *k,* and *OFC*(*k*) is the subject’s OFC activity for trial *k*. The model parameters *a*_*c*_ and *γ*_*c*_ are fitted using maximum likelihood estimation for each card separately^29^. The coefficients *γ*_*c*_ for *c* = {2, 4, 8, 10} were found to be not significant, indicating that OFC activity only statistically significantly biases behavior during the ambiguous trials on the 6-card. The *p*-values associated with OFC influence on 2, 4, and 8 cards saturate to 1, and the *p*-value for 10-card trials is 0.18 (after applying a Bonferroni correction to compensate for the four comparisons).

**Figure 3:**
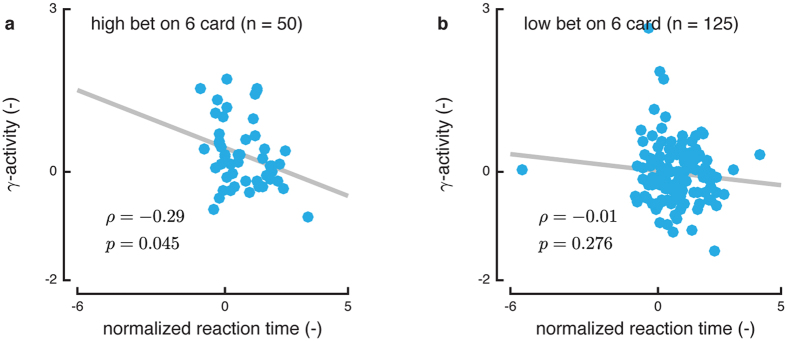
Reaction time and OFC activity correlations for high (**a**) and low (**b**) bets for 6 cards. For high bets, there is a negative and significant correlation between OFC activity and reaction time, meaning that higher activity resulted in faster decisions. No significant correlation was observed in the low bet scenario. The number *n* denotes the number of trials pooled across patients.

So far, effects of OFC bias on behavior have only been assessed through bet decisions. However, another key aspect of behavior in this task is reaction time. If OFC activity biases towards the decision made, then a faster reaction time would be expected when the OFC activity is higher; while if the opposite were true, a slower reaction time would be expected. This relationship between normalized reaction times and OFC power was tested for both high and low bet trials for 6 cards using Pearson’s correlation tests (Fig. 3). The correlation was only significant for the case of the high bets. Here, higher OFC activity, which biases towards high bets, is correlated with faster reaction times, as expected. The lack of significance for the low bet cases may suggest that OFC only selectively influences decision-making. This type of selective influence of other brain areas has been noted in other decision modalities. For example, the insular cortex selectively activates for risk-averse choices while the nucleus accumbens selectively activates for risk-seeking choices^30^.

### Linking OFC to previous outcomes

The next step was to assess how this OFC activity linked back to outcomes of previous trials, as this may impact how the subject “feels” on a trial-by-trial basis. While not a direct measure of emotional state, we used cumulative past trial outcomes as a proxy. It was also assumed that subjects have an accurate perception of the odds of winning given a specific card. The overall behavior shown in Fig. 1b supports this. Specifically, we constructed a fading memory model of cumulative mismatched expectations that we herein refer to as “luck” *L*(*k*) on trial *k*. The luck variable updates as follows:


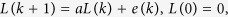


where *a* is a decay factor (0 ≤ *a* ≤ 1) and *e*(*k*) is the mismatched expectations on trial *k*, that is, the difference between the actual outcome (loss = −1, draw = 0, or win = 1 and expected outcome given the player card *c*(*k*) (computed as 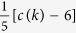). Note that *e*(*k*) enters the model only during trials where expectations are mismatched. Further note how the mismatched expectations, *e*(*k*), functionally acts similarly to the error signal in reinforcement learning, as it tracks the mismatch between actual and expected outcome^31^ However, this analysis differs from traditional reinforcement learning as the changes in behavior are not a function of the subject’s previous actions. This focus on mismatched outcomes is in part motivated by the previously mentioned theory on reversal learning proposed in ref. 10.

We hypothesize that luck is linked to OFC activity on a trial-by-trial basis. To test this hypothesis, we varied the decay factor *a* between 0 and 1 in 0.01 increments and computed the Pearson’s correlation coefficient between luck and OFC activity. The optimal decay factor was *a* = 0.97 (*ρ* = 0.13, *p* = 0.043) after a Bonferroni correction). This optimal decay factor was used for the remainder analyses. To further examine the correlation between OFC activity and luck, we separated the trials between high-luck and low-luck conditions (defined as the bottom third and top third of the values taken by luck variable for all patients) and computed the average normalized spectrograms for both conditions. High-luck trials showed higher OFC oscillatory power than low-luck trials (Fig. 2b, first and second panels). Interestingly, the cluster-based nonparametric statistical test identified a significant cluster (*p* = 0.040) in the time-frequency vicinity of the cluster identified when separating trials based on high-bet and low-bets conditions on 6-card trials (Fig. 2b, third panel).

Now, knowing the link between luck and OFC activity, we tested the hypothesis that luck predicted betting decisions by modeling the probability to bet high on 6-card trials as a function of luck:


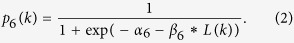


The fitted model showed that luck significantly influenced betting decisions (*β*_6_ = 0.20, *p* = 0.028). This indicates positive luck biases subjects to bet high on 6 cards.

By linking OFC activity, luck, and decision-making together, a possible confound is introduced. Does OFC activity actually influence future decisions or does it merely encode luck? To assess this confound, another model was used that simultaneously accounts for both the influence of luck and OFC activity on 6-card betting decisions:


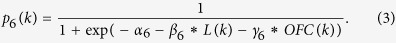


After including both luck and OFC as covariates to explain the variability in 6-card bets around the baseline, only the OFC activity remains statistically significant in the model (γ_6_ = 0.85, *p* = 0.004), while luck is no longer significant (*β*_6_ = 0.17, *p* = 0.065). The point of the final GLM that we construct in equation (3) is to show the data suggests OFC activity carries much of the same information about betting behavior as luck and that both are redundant in the probability model. The redundancy does not refute the hypothesis that the OFC activity encodes the emotional state that then drives betting behavior, and is not surprising since we already showed that OFC activity and luck are correlated.

### Linking previous outcomes to emotional state

In order to clarify luck’s link to the subject’s emotional state, we compared the signal *L* to each subject’s HRV signal, which is an objective measure of a person’s emotional state^32^. Specifically, it measures the effects of both parasympathetic and sympathetic systems on the subject^32^. HRV for each trial was calculated from the variance of the interbeat intervals preceding that trial and was measured through concurrently recorded ElectroCardioGrams (ECG)^33^,^34^. HRV was found to be significantly correlated with luck (*ρ* = −0.09, *p* = 0.039). This result suggests that luck is an appropriate proxy to emotional processing. See *Heart Rate Variability* in *Methods* for further details on the calculation and analysis.

## Discussion

In this study, we find that OFC activity encodes an internal bias that is predictive of betting behavior on ambiguous trials where simple reason (e.g., maximizing expected reward) cannot drive the decision; and, this activity also correlates with reaction times of bets when subjects bet “high” on these trials. Then, we show that this OFC “bias” signal correlates to previous outcomes of mismatched expectations which are taken as a proxy to the subject’s emotional state. Finally, we show that cumulative mismatched expectations correlate to HRV, which is an objective measure of emotional state.

As mentioned above, we observe OFC activity that precedes any task information biases future decisions. Looking at the OFC as a biasing agent helps shed new light on the exact role of the OFC in the decision-making process. Two popular theories for the role of the OFC are response inhibition and the emotion marker theory. For the former theory, lesions interfere with reversal learning as well as go/no-go regulation^35^. Additionally, patients with obsessive-compulsive disorder have been linked to having a dysfunctional OFC^11^. However, response inhibition can be produced in some scenarios even with OFC damage, so this likely is not its fundamental role^36^.

For the emotional marker theory, OFC lesions are associated with an inability to link past emotions to future decisions^37^. Specifically, subjects with lesions performing the Iowa gambling task still had somatic markers associated with making risky choices but never ended up being dissuaded against them^14^. More recent work, however, has shown that this influence is only prevalent in situations with model-based learning as opposed to more heuristically-derived choices^37^.

Both of these theories make sense in the new context of bias encoding as a result of mismatched expectations. A dysfunctional OFC can no longer regulate bias correctly. This results in dysfunctional biasing of behavior that could manifest as failure to adapt and failure to control compulsions. These deficits can be linked to both ideas of emotional processing and response inhibition. It also answers key concerns for both theories. This biasing occurs as a result of mismatches between realizations and expectations from previous trials, which can only be present when there is an expectation in place. This could explain why learning and response inhibition are possible in situations that lack prior training, despite OFC lesions.

While the results described here can be put in the context of prior theories of OFC function, they fit most cleanly into the recent description of the OFC as containing a cognitive map^19^. The bias signal observed in the OFC aligns with current ideas that the OFC reflects the location of the organism within task relevant state-space. It seems to reflect an internal state, whether tied to memory or emotion, that helps explain differences in behavioral responses that are not reducible to difference in trial-specific external stimuli^20^,^21^.

The effects of the external environment on decision-making can be clearly seen in the predictable behavior of subjects when given low (2-card, 4-card) or high (8-card, 10-card) cards. However, after those effects are explained away, the question of why subjects sometimes bet high and sometimes bet low on the intermediate 6-card remains. The same stimulus leads to a differing response across trials. In this case, we proposed the existence of a hidden state, perhaps emotional, existing before the trial that would bias the subject toward betting high or low in ambiguous situations. When we treat a portion of the power spectrum of the OFC as an estimate of the hidden state, the predictions of betting behavior are improved. Interesting parallels exist with free-choice trials (similar to 6-card trials) in odor-discrimination tasks where ventral tegmental area (VTA) dopamine neurons no longer fire before the reward is acquired when the OFC has been lesioned^20^. In both cases, behavior is driven by an internal state rather than by an external cue. This state-space interpretation of OFC function has clear parallels with advances in control theory frequently employed in modern engineering, perhaps indicating a convergence between neuroscience and engineering theory.

## Methods

### Subjects

At the Cleveland Clinic, patients with medically intractable epilepsy may undergo SEEG recording in order to localize the seizure focus. In this study, aside from the behavioral experiments, no alterations were made to the patient’s clinical care including the placement of the electrodes^24^. All experimental protocols were approved by the Cleveland Clinic Institutional Review Board. Methods were carried out in accordance with the approved guidelines by the Cleveland Clinic Institutional Review Board. Subjects enrolled voluntarily and gave informed consent under criteria approved by the Cleveland Clinic Institutional Review Board. A total of five subjects volunteered to perform the task that had SEEG electrodes in the OFC. Details on these recordings and eventual resections of these five patients are noted in Table 1.

Subjects were implanted with 10 to 14 depth electrodes. Implantation was performed using robot-assisted surgery along with co-registered fMRI and angiograms to ensure safe implantation^23^. Once inserted, SEEG electrophysiological data were acquired using a Nihon Kohden 1200 EEG diagnostic and monitoring system (Nihon Kohden America, USA) at a sampling rate of 1 or 2 kHz. Behavioral data were simultaneously acquired through the MonkeyLogic Matlab toolbox^27^.

There are standard concerns in analyzing data from epileptic patients. First, patients are often on medication, which might affect the neurophysiology of the brain. For clinical purposes, patients were kept off of their anti-seizure medication for their entire stay at Cleveland Clinic, so these effects would be minimized. Secondly, actual seizures might impact the neurophysiology around the seizure focus. Human epilepsy recordings are taken to localize the seizure focus, so overlap is expected between seizure focus and areas recorded. We only had one subject whose seizure focus overlapped with OFC, as indicated in Table 1.

### Gambling Task

The gambling task (Fig. 1a) is based on a simple game of high card where subjects would win virtual money if their card beat the computer’s card. Specifically, in the beginning of each trial, the subject controls a cursor via a planar manipulandum to a fixation target. During fixation, subjects must center the cursor in less than 8 seconds. Once centered, the subject is shown his card^2,4,6,8^ or^10^ for a duration of 2 seconds. The card is randomly chosen with equal distribution. The computer’s card is initially hidden. The screen then shows the two possible choices: a high bet ($20) or a low bet ($5). The subject has 6 seconds to select one with his cursor. Following selection, the computer’s card, which follows the same distribution, is revealed. If the computer’s card is larger than the player’s card, then the subject loses the amount he bets. If the computer’s card is smaller than the player’s card, then the subject wins the amount he bet. If the two cards are equal, then no virtual money is won or lost. The final screen depicts the amount won or lost.

### Spectral Analysis

Oscillatory power was calculated using multitapers from the Chronux toolbox^27^. Three orthogonal tapers were used with a 300 ms window sliding at 50 ms steps. Frequencies under 10 Hz were dropped because of the Rayleigh criterion and analyzed upwards to 100 Hz. Afterwards, each frequency bin’s power was normalized based on the power across the entire recording session by fitting the log of the power in each frequency bin to a standard normal distribution. The mean and standard deviation used for the normalization were computed from the power between the 1^th^ and 95^th^ percentiles of the data set. This calculation was performed for every electrode’s recording with the final normalized power being averaged across all OFC electrodes. In addition, we removed artifacts by identifying time points in the spectrograms for which the median of the absolute value of the power across all frequencies is larger than 2.5. Finally, in order to remove the effect of 60 Hz power-line noise, we ignored the frequency bins between 56.66 Hz and 63.33 Hz in all analyses.

Due to our hypothesis that there exists an internal bias before any stimulus is shown, we focused our. analysis on a time window preceding the Show Card epoch.

### Cluster-Based Nonparametric Statistical Test

We used a cluster-based nonparametric statistical test to leverage the dependency between adjacent time-frequency windows in order to avoid over-penalizing with multiple comparison corrections^28^. For each window in the spectrogram, a null distribution was created by shuffling the condition labels 5000 times between trials within each subject. Within each shuffle, we computed a *t*-statistic and a *p*-value for each window of the newly labeled spectrograms (independent two-sample *t*-test with both tails, unequal sample sizes, and unequal variances). Clusters were formed by grouping windows with significant *p*-values (<0.05) that were adjacent in either time or frequency. The cluster-level test statistic was calculated by taking the sum of absolute values of the *t*-statistics for each window in the cluster. This prioritizes clusters that both have strong differences as well as large sizes. A null distribution of cluster statistics was created using the same process but with the 5000 spectrograms obtained from the originally shuffled labels. The observed cluster statistic was then compared against this null distribution of cluster statistics in order to obtain the final *p*-value of the test. Data from all patients were pooled together but the labels were permuted within each subject only.

### Correlating OFC Activity and Reaction Time

We normalized reaction times for each patient separately by fitting the log of the reaction times to a standard normal distribution. We computed a Pearson’s correlation coefficient between the OFC activity and the normalized reaction time for all high-bet trials on 6 cards and all low-bet trials on 6 cards, respectively. The data from all patients was pooled together.

### Correlating OFC Activity and Luck

To test whether OFC activity is linked to luck, we computed a Pearson’s correlation coefficient between the OFC activity and the fading memory luck variable. In order to avoid the correlation being biased toward stronger values by the earliest trials for which the luck is dominated by a few trials’ worth of outcome-prediction error (initial transient effects), the first 10 trials for each subject were ignored. Data from all patients were pooled together.

Other models of luck were considered. However, the model discussed in this paper was selected based on its alignment with past literature on the subject^38^,^39^,^40^, its intuitive nature, and the fact that it provides the highest correlation with OFC activity.

### Generalized Linear Model Regression of Betting Behavior

We estimated the coefficients for each generalized linear regression of the responses (high and low bets) on the predictors (OFC activity and/or luck variable) using a binomial distribution and a logistic link function. When needed, we estimated these regressions for each card separately. The data from all patients was pooled together.

### Correlating Luck and Heart Rate Variability

We had concurrently recorded ECG for 4 of the 5 subjects. The R peaks in the ECG signal were detected using the Pan-Tompkins algorithm^33^. HRV was calculated by taking the variance of interbeat intervals measured as the time between R peaks. The HRV for each trial was taken over a 5-minute window preceding the show card epoch. This window length is the established standard for HRV recording^34^. The HRV was normalized within each subject by dividing by the mean HRV across all trials and then by taking the logarithm as HRV distributions are often skewed right. We computed a Pearson’s correlation coefficient between the normalized HRV and the fading memory luck variable. As in the case of the correlation between luck and OFC activity, the first 10 trials for each subject were ignored to avoid initial transient effects. Data from all patients were pooled together.

### Limitations of our Study

Finally, we discuss the limitations of this study.

We had relatively few patients performing our decision-making task. This is because not all patients consented and/or met the criteria of our study. The small sample size of the study population is further limited by the fact that each patient had electrodes implanted in different brain regions. In order to compensate for this small sample size, we pooled the trials over the subjects. In addition, we studied the sensitivity of the observed effects to the inclusion of each patient in the analysis: rerunning the cluster analysis leaving each subject out one at a time produces a cluster in the same time-frequency regions. This sensitivity analysis suggests that the effect truly emerges from *all* patients and not only from one patient (see Supplementary Information).

## Additional Information

**How to cite this article**: Sacré, P. *et al*. Lucky Rhythms in Orbitofrontal Cortex Bias Gambling Decisions in Humans. *Sci. Rep.* **6**, 36206; doi: 10.1038/srep36206 (2016).

**Publisher’s note**: Springer Nature remains neutral with regard to jurisdictional claims in published maps and institutional affiliations.

## Supplementary Material

Supplementary Information

## Figures and Tables

**Table 1:** Study subject’s characteristics.

ID	Gender	Age [yr.]	Epilepsy Duration [yr.]	Seizure Focus	Trials (6/all)
1	female	41	38	Lateral Temporal Lobe (R)	39/162
				Amygdala (R)	
				Hippocampus (R)	
2	female	31	15	Temporal Lobe (R)	34/144
				Amygdala (R)	
				Hippocampus (R)	
3	female	53	23	Orbitofrontal Lobe (R)	35/136
				Temporal Lobe (R)	
4	female	32	13	Not-identified (no surgery)	33/154
5	male	28	11	Lateral Temporal Lobe (R)	34/182
				Amygdala (R)	
				Hippocampus (R)	

This table provides clinically relevant information on each of the subjects: the gender, the age in years, the duration of epilepsy in years, and the seizure focus. The seizure focus encompasses the areas that were resected from after SEEG recordings. All of these areas were from the right hemisphere, as indicated by the “R”s. In addition, it provides the number of trials recorded for 6 cards as well as for all cards.

## References

[b1] Kacelnik A. & Bateson M. Risk-sensitivity: crossroads for theories of decision-making. Trends in Cognitive Sciences 1(8), 304–309 (1997). 21223933 10.1016/S1364-6613(97)01093-0

[b2] Tom S. M., Fox C. R., Trepel C. & Poldrack R. A. The Neural Basis of Loss Aversion in Decision-Making Under Risk. Science 315(5811), 515–518 (2007). 17255512 10.1126/science.1134239

[b3] Kim H., Choi M. & Jang I. Lateral OFC Activity predicts decision bias due to first impressions during ultimatum games. Journal of Cognitive Neuroscience 24(2), 428–439 (2011). 21942764 10.1162/jocn_a_00136

[b4] Pfister H. R. & Böhm G. The multiplicity of emotions: A framework of emotional functions in decision making. Judgment and decision making 3(1), 5–17 (2008).

[b5] Damasio A. R. Descartes’ Error: Emotion, Reason, and the Human Brain (Grosset/Putnam, New York, NY, 1994).

[b6] Lerner J. & Keltner D. Beyond valence: Toward a model of emotion-specific influences on judgement and choice. Cognition and Emotion 14(4), 473–493 (2000).

[b7] Padoa-Schioppa C. & Assad J. A. Neurons in the orbitofrontal cortex encode economic value. Nature 441(7090), 223–226 (2006). 16633341 10.1038/nature04676 PMC2630027

[b8] Levy D. J. & Glimcher P. W. The root of all value: a neural common currency for choice. Current Opinion in Neurobiology 22(6), 1027–1038 (2012). 22766486 10.1016/j.conb.2012.06.001 PMC4093837

[b9] Camille N., Tsuchida A. & Fellows L. K. Double dissociation of stimulus-value and action-value learning in humans with orbitofrontal or anterior cingulate cortex damage. The Journal of Neuroscience 31(42), 15048–15052 (2011). 22016538 10.1523/JNEUROSCI.3164-11.2011 PMC6623552

[b10] Schoenbaum G., Saddoris M. P. & Stalnaker T. A. Reconciling the roles of orbitofrontal cortex in reversal learning and the encoding of outcome expectancies. Annals of the New York Academy of Sciences 1121, 320–335 (2007). 17698988 10.1196/annals.1401.001 PMC2430624

[b11] Chamberlain S. R. . Orbitofrontal dysfunction in patients with obsessive-compulsive disorder and their unaffected relatives. Science 321(5887), 421–422 (2008). 18635808 10.1126/science.1154433

[b12] Bohn I., Giertler C. & Hauber W. Orbital prefrontal cortex and guidance of instrumental behavior in rats under reversal conditions. Behavioral Brain Research 143(1), 49–56 (2003). 10.1016/s0166-4328(03)00008-1 12842295

[b13] Kim J. & Ragozzino M. E. The involvement of the orbitofrontal cortex in learning under changing task contingencies. Neurobiology of Learning and Memory 83, 125–133 (2005). 15721796 10.1016/j.nlm.2004.10.003 PMC3206595

[b14] Bechara A. The role of emotion in decision-making: evidence from neurological patients with orbitofrontal damage. Brain and Cognition 55(1), 30–40 (2004). 15134841 10.1016/j.bandc.2003.04.001

[b15] Walton M. E., Behrens T. E. J., Buckley M. J., Rudebeck P. H. & Rushworth M. F. S. Separable Learning Systems in the Macaque Brain and the Role of Orbitofrontal Cortex in Contingent Learning. Neuron 65(6), 927–939 (2010). 20346766 10.1016/j.neuron.2010.02.027 PMC3566584

[b16] Wilson R. C., Takahashi Y. K., Schoenbaum G. & Niv Y. Orbitofrontal cortex as a Cognitive Map of Task Space. Neuron 81(2), 267–279 (2014). 24462094 10.1016/j.neuron.2013.11.005 PMC4001869

[b17] Camille N., Griffiths C. A., Vo K., Fellows L. K. & Kable J. W. Ventromedial frontal lobe damage disrupts value maximization in humans. The Journal of Neuroscience 31(20), 7527–7532 (2011). 21593337 10.1523/JNEUROSCI.6527-10.2011 PMC3122333

[b18] Luk C. H. & Wallis J. D. Choice Coding in Frontal Cortex during Stimulus-Guided or Action-Guided Decision-Making. The Journal of Neuroscience 33(5), 1864–1871 (2013). 23365226 10.1523/JNEUROSCI.4920-12.2013 PMC3711610

[b19] Stalnaker T. A., Cooch N. K. & Schoenbaum G. What the orbitofrontal cortex does not do. Nature Neuroscience 18, 620–627 (2015). 25919962 10.1038/nn.3982 PMC5541252

[b20] Takahashi Y. K. . Expectancy-related changes in firing of dopamine neurons depend on orbitofrontal cortex. Nature Neuroscience 14, 1590–1597 (2011). 22037501 10.1038/nn.2957 PMC3225718

[b21] Kennerley S. W. & Walton M. E. Decision making and reward in frontal cortex: complementary evidence from neurophysiological and neuropsychological studies. Behavioral Neuroscience 125(3), 297–317 (2011). 21534649 10.1037/a0023575 PMC3129331

[b22] Logothetis N. K. What we can do and what we cannot do with fMRI. Nature Reviews 453(7197), 869–878 (2008). 10.1038/nature06976 18548064

[b23] Öngür D. & Price J. L. The organization of Networks within the Orbital and Medial Prefrontal Cortex of Rats, Monkeys and Humans. Cerebral Cortex 10(3), 206–219 (2000). 10731217 10.1093/cercor/10.3.206

[b24] Johnson M. . Performing behavioral tasks in subjects with intracranial electrodes. Journal of Visualized Experiments (92), e51947 (2014). 25349952 10.3791/51947 PMC4672964

[b25] Cossu M. . Stereoelectroencephalography in the presurgical evaluation of focal epilepsy: a retrospective analysis of 215 procedures. Neurosurgery 57(4), 706–718 (2005). 16239883

[b26] Ward L. M. Synchronous neural oscillations and cognitive processes. Trends in Cognitive Sciences 7(12), 553–559 (2003). 14643372 10.1016/j.tics.2003.10.012

[b27] Bokil H., Andrews P., Kulkarni J., Mehta S. & Mitra P. Chronux: a platform for analyzing neural signals. Journal of Neuroscience Methods 191(1), 146–151 (2010). 10.1016/j.jneumeth.2010.06.020 PMC2934871 20637804

[b28] Maris E. & Oostenveld R. Nonparametric statistical testing of EEG- and MEG-data. Journal of Neuroscience Methods 164(1), 177–190 (2007). 17517438 10.1016/j.jneumeth.2007.03.024

[b29] McCullagh P. & Nelder A. Generalized linear models, 2nd ed. (Chapman & Hall, London, UK, 1989).

[b30] Kuhnen C. M. & Knutson B. The neural basis of financial risk taking. Neuron 47(5), 763–770 (2005). 16129404 10.1016/j.neuron.2005.08.008

[b31] Holroyd C. M. & Coles M. G. H. The neural basis of human error processing: reinforcement learning, dopamine, and the error-related negativity. Psychological Review 109(4), 679–709 (2002). 12374324 10.1037/0033-295X.109.4.679

[b32] Appelhans B. M. & Luecken L. J. Heart rate variability as an index of regulated emotional responding. Review of General Psychology 10(3), 229–240 (2006).

[b33] Pan J. & Tompkins W. J. A real-time QRS detection algorithm. IEEE Transactions on Biomedical Engineering BME-32(3), 230–236 (1985). 10.1109/TBME.1985.325532 3997178

[b34] Gritti I. . Heart Rate Variability, Standard of Measurement, Physiological Interpretation and Clinical Use in Mountain Marathon Runners during Sleep and after Acclimatization at 3480 m. Journal of Behavioral and Brain Science 3(1), 26–48 (2013).

[b35] Horn N., Dolan M., Elliott R., Deakin J. & Woodruff P. Response inhibition and impulsivity: an fMRI study. Neuropsychologia 41(14), 1959–1966 (2003). 14572528 10.1016/s0028-3932(03)00077-0

[b36] Chudasama Y., Kralik J. D. & Murray E. A. Rhesus monkeys with orbital prefrontal cortex lesions can learn to inhibit prepotent responses in the reversed rewards contingency task. Cerebral Cortex 17(5), 1154–1159 (2007). 16774961 10.1093/cercor/bhl025

[b37] McDannald M. . Model-based learning and the contribution of the orbitofrontal cortex to the model-free world. European Journal of Neuroscience 35(7), 991–996 (2013). 10.1111/j.1460-9568.2011.07982.x PMC3529907 22487030

[b38] O’Doherty J. P., Dayan P., Friston K., Critchley H. & Dolan R. J. Temporal Difference Models and Reward-Related Learning in the Human Brain. Neuron 38(2), 329–337 (2003). 12718865 10.1016/s0896-6273(03)00169-7

[b39] O’Doherty J. P. . Dissociable Roles of Ventral and Dorsal Striatum in Instrumental Conditioning. Science 304(5669), 452–454 (2004). 15087550 10.1126/science.1094285

[b40] O’Doherty J. P., Hampton A. & Kim H. Model-Based fMRI and Its Application to Reward Learning and Decision Making. Annals of the New York Academy of Sciences 1104(1), 35–53 (2007). 17416921 10.1196/annals.1390.022

