# Erratum: Lucky Rhythms in Orbitofrontal Cortex Bias Gambling Decisions in Humans

**DOI:** 10.1038/srep42830

**Published:** 2017-02-16

**Authors:** Pierre Sacré, Matthew S. D. Kerr, Kevin Kahn, Jorge Gonzalez-Martinez, Juan Bulacio, Hyun-Joo Park, Matthew A. Johnson, Susan Thompson, Jaes Jones, Vikram S. Chib, John T. Gale, Sridevi V. Sarma

Scientific Reports 6: Article number: 3620610.1038/srep36206; published online: 11 10 2016; updated: 02 16 2017

Affiliation 1 was incorrectly listed as ‘Department of Biomedical Engineering, Johns Hopkins University, Baltimore, MD 21211, USA’ in the original version of the Article. The correct affiliation is listed below:

Department of Biomedical Engineering, Johns Hopkins University, Baltimore, MD 21218, USA.

In addition, in the Introduction section,

“Afterwards, subjects are shown their card^2,4,6,8^ or^10^ that is randomly chosen with equal distribution (known by the subjects)”.

now reads:

“Afterwards, subjects are shown their card (2, 4, 6, 8 or 10) that is randomly chosen with equal distribution (known by the subjects).”

In the Methods section under subheading ‘Gambling Task’,

“Once centered, the subject is shown his card^2,4,6,8^ or^10^ for a duration of 2 seconds”.

now reads:

“Once centered, the subject is shown his card (2, 4, 6, 8 or 10) for a duration of 2 seconds”.

These errors have now been corrected in the HTML and PDF versions of this Article.

