# Supplementary material for: Lucky Rhythms in Orbitofrontal Cortex Bias Gambling Decisions in Humans
Source: Sci Rep. 2016 Nov 10;6:36206. doi: 10.1038/srep36206 (PMC5103224; doi:10.1038/srep36206)
Supplement: Supplementary Information [file srep36206-s1.pdf]

Supplementary Information:
Lucky Rhythms in Orbitofrontal Cortex Bias Gambling Decisions in Humans

Pierre Sacré[1,*], Matthew S.D. Kerr[1,*], Kevin Kahn[1], Jorge Gonzalez-Martinez[2], Juan Bulacio[2], Hyun-Joo Park[3], Matthew A. Johnson[3], Susan Thompson[2], Jaes Jones[3], Vikram S. Chib[1], John T. Gale[3,4,†], Sridevi V. Sarma[1,†,‡]

[1]Department of Biomedical Engineering, John Hopkins University, Baltimore, MD

[2]Center for Epilepsy, Neurological Institute, Cleveland Clinic, Cleveland, OH

[3]Department of Neuroscience, Lerner Research Institute, Cleveland Clinic, Cleveland, OH

[4]Center for Neurological Restoration, Neurological Institute, Cleveland Clinic, Cleveland, OH

[*]Co-first author, [†]Equal last author, [‡]Correspondence to Sridevi V. Sarma (ssarma2@jhu.edu)

Control Tests on Modulation of Orbitofrontal Cortex Oscillatory Power Before Show Card

We tested if the modulation of OrbitoFrontal Cortex (OFC) oscillatory power in the time period preceding the Show Card of 6-card trials was merely a result of other effects than an internal bias. We used the nonparametric cluster test in order to investigate the modulation of OFC activity based on

1. win and loss conditions on the previous trial (see Supplementary Figure 1),
2. low-bet-and-win and high-bet-and-loss conditions on the previous trial (see Supplementary Figure 2), and
3. high and low reward-prediction error on previous trial (see Supplementary Figure 3).

No significant clusters were found using our cluster-based statistical test.

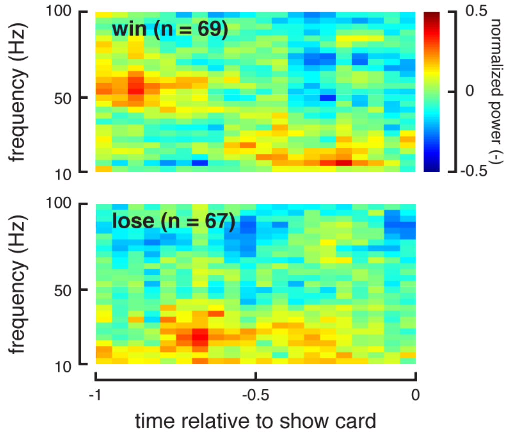

Supplementary Figure 1. Control test based on win and loss conditions on the previous trial. Separating data preceding the Show Card on the 6-card trials by win and lose conditions on the previous trial shows no significant difference in their average spectrograms. No cluster was detected by the statistical test. The number n denotes the number of trials pooled across patients.

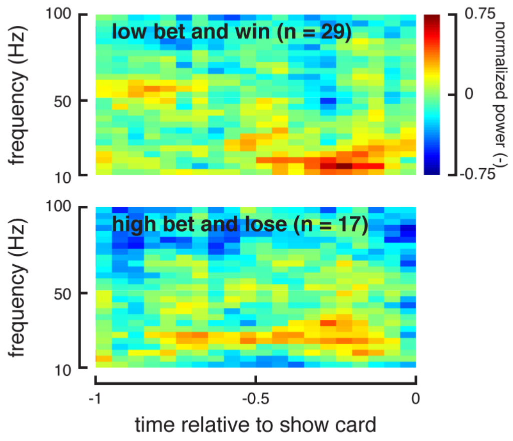

Supplementary Figure 2. Control test based on low-bet-and-win and high-bet-and-lose conditions on the previous trial. Separating data preceding the Show Card on the 6-card trials by low-bet-and-win and high-bet-and-lose conditions on the previous trial shows no significant difference in their average spectrograms. No cluster was detected by the statistical test. The number n *denotes the number of trials pooled across patients.*

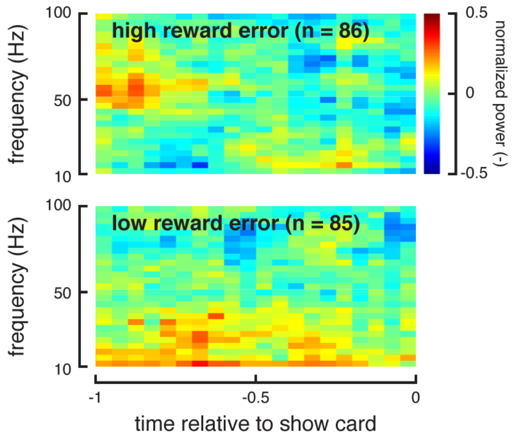

Supplementary Figure 3. Control test based on high and low reward-prediction error conditions on the previous trial. Separating data preceding the Show Card on the 6-card trials by high-reward-prediction-error (positive) and low-reward-prediction-error (negative) conditions on the previous trial shows no significant difference in their average spectrograms. No cluster was detected by the statistical test. The number n *denotes the number of trials pooled across patients.*

Sensitivity Analysis

The behavior of all patients (including subject 3) follows the same trend. In the following figure, we show the average percentage of high bets across cards for each patient individually (see Supplementary Figure 4).

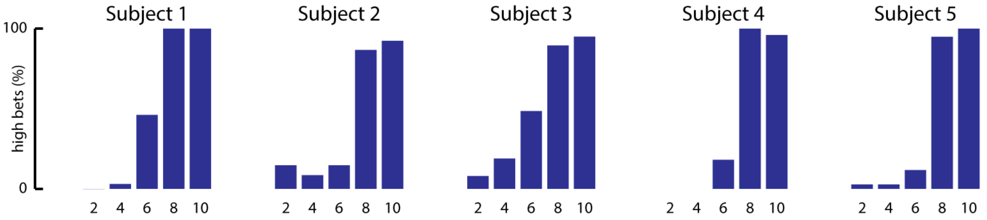

Supplementary Figure 4. Individual average bet decisions across cards. The behavior of all patients (including subject 3) follows the same trend.

Looking at the spectrograms for each patient and each OFC electrode contact in high bet and low bet 6-card trials (and their differences) also suggests that the effect is not only present in subject 3 (data not shown).

To further quantify our observations of the spectrograms, we reran the cluster analysis leaving each subject out, one at a time. If we leave out subject 3 (whose OFC was resected), and rerun the cluster analysis, we still observe a strong cluster; however, the *p*-value increases from 0.0418 to 0.1074. With this said, we get similar results when we remove other subjects as well. Indeed, a strong cluster persists in roughly the same time-frequency region when we remove each subject one by one (see Supplementary Figure 5). Interestingly, when we remove subjects 1 or 4, the cluster is still significant ($p < 0.05$), and when we remove patients 2, 3 or 5, we lose significance. In fact, the most significant change is when we remove subject 2 and not subject 3. While distributed among subjects, the effect is largest in subject 2 (a subject with no relevant epileptogenic zone concerns). This also supports the hypothesis the trend is not driven by epileptogenic zone recordings. This sensitivity is definitely due to our small sample size.

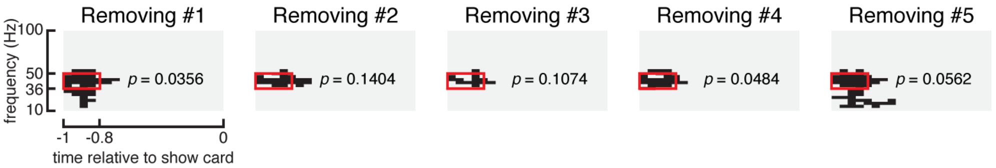

Supplementary Figure 5. Sensitivity analysis of the cluster analysis to inclusion of patients. A strong cluster persists in roughly the same time-frequency region when we remove each subject one by one. The red rectangle delimits the time-frequency region of interest as defined by our initial cluster including 5 patients.
